# The Standard Dictionary of Funk & Wagnalls

**Published:** 1894-06

**Authors:** 


THE STANDARD DICTIONARY of Funk & Wagnalls was reviewed in the April number of this journal at page 122. It should have been stated that the dictionary is only sold by subscription and when completed will be bound in either one or two volumes as desired. The following are the prices: Single-volume edition: half Russia, $12.00; full Russia, $14.00; full Morocco, $18.00. Two-volume edition: half-Russia, per volume, $7.50; per set, $15.00; full Russia, per volume, $8.50; per set, $17.00; full Morocco, per volume, $11.00; per set, $22.00. The full-bound books all have Denison's Patent Reference Index. Funk & Wagnalls Company. New York: 18-20 Astor Place. London: 44 Fleet Street. Toronto; 11 Richmond Street W.

